# Trombose venosa profunda e vírus chicungunha

**DOI:** 10.1590/1677-5449.009616

**Published:** 2017

**Authors:** Marcos Arêas Marques, Fernanda Penza Adami de Sá, Otília Lupi, Patricia Brasil, Arno von Ristow

**Affiliations:** 1 Universidade do Estado do Rio de Janeiro – UERJ, Departamento de Angiologia, Rio de Janeiro, RJ, Brasil.; 2 Hospital Universitário Pedro Ernesto, Departamento de Angiologia, Rio de Janeiro, RJ, Brasil.; 3 Fundação Oswaldo Cruz – FIOCRUZ, Instituto Nacional de Infectologia Evandro Chagas, Rio de Janeiro, RJ, Brasil.; 4 Pontifícia Universidade Católica do Rio de Janeiro – PUC-Rio, Departamento de Cirurgia Vascular, Rio de Janeiro, RJ, Brasil.

**Keywords:** vírus chicungunha, febre de chicungunha, trombose venosa profunda

## Abstract

Algumas infecções virais sistêmicas podem estar relacionadas ao desenvolvimento de trombose venosa profunda e/ou embolia pulmonar. Essa associação já está bem descrita em pacientes com infeções pelo vírus da imunodeficiência humana (HIV), hepatite C ou influenza. Recentemente introduzido no continente americano, o vírus chicungunha, agente etiológico da febre de chicungunha, ainda não tem essa relação bem sedimentada, mas com o aumento progressivo de sua incidência e pelo fato dessa infecção causar, muitas vezes, uma restrição severa da locomoção por poliartralgia e uma possível lesão endotelial direta, casos de tromboembolismo venoso podem começar a ser descritos. Neste relato de caso, descrevemos um paciente que desenvolveu trombose de veia poplítea direita durante internação para tratamento de febre por infecção por vírus chicungunha e poliartralgia severa.

## INTRODUÇÃO

A febre de chicungunha (FC) é causada pelo vírus chicungunha (CHIKV), um a*lphavirus* transmitido aos humanos através da picada de mosquito do gênero *Aedes,* inicialmente descrito nos anos 1950 na África central e posteriormente em outros países africanos e na Ásia^1^. Em 2007, o CHIKV chegou à Europa, causando um surto de FC na Itália^1^, e isso provavelmente provocou o movimento do CHIKV em direção a novos territórios, como Austrália e hemisfério oeste^1^. Em dezembro de 2013, a Organização Pan-americana de Saúde emitiu um alerta de transmissão autóctone de CHIKV nas Américas pela primeira vez^2^.

Clinicamente, a FC se caracteriza por início abrupto de febre alta (> 38,9 °C), calafrios e fotofobia, que normalmente duram sete dias, associada, na maioria dos casos, a poliartralgia severa, usualmente simétrica, que acomete quadril, cotovelos, dedos, joelhos e tornozelos, limitando a locomoção do paciente por meses^1^^,^ 2.

Eventualmente, o paciente pode apresentar exantema maculopapular em tronco, face e extremidades, prurido, cefaleia, fadiga, náuseas, vômitos, conjuntivite, linfadenopatia cervical e mialgia^1^^,^ 2. Laboratorialmente, a FC se caracteriza agudamente por leucopenia, trombocitopenia, hipocalemia e elevação leve a moderada das transaminases hepáticas^1^. As complicações agudas mais comuns são secundárias ao acometimento do sistema nervoso central, como a síndrome de Guillain-Barré e ocular, como a uveíte e retinite^1^. Cronicamente, é comum o desenvolvimento de síndromes articulares, com persistência de poliartrite em 30-40% dos casos^2^. O acometimento vascular na FC é pouco descrito e normalmente restrito ao fenômeno de Raynaud persistente após a sua fase aguda^1^. Abaixo, descrevemos o caso de um paciente que desenvolveu trombose de veia poplítea direita durante internação para tratamento de FC e poliartralgia severa.

## DESCRIÇÃO DO CASO

Paciente do sexo masculino, 55 anos de idade, caucasiano, ex-tabagista, procurou o pronto-atendimento (PA) com história de febre (> 39 °C), astenia, cefaleia e artralgia de joelhos e ombro direito há três dias. Recebeu alta hospitalar com orientação para se manter hidratado e prescrição de paracetamol. Três dias após, procurou novamente o PA com relato de manutenção da febre elevada, astenia e cefaleia, piora da intensidade da artralgia, que passou a acometer, também, os tornozelos, e dor em panturrilha esquerda associada a edema bilateral de tornozelos, mais evidente à esquerda. Os exames laboratoriais de emergência iniciais demonstravam trombocitopenia moderada (108.000 plaquetas), leucopenia (3.800 leucócitos) e hematócrito normal (41%). Devido à intensidade da febre e da poliartralgia e ao edema assimétrico de membros inferiores, o paciente foi internado para controle sintomático e investigação diagnóstica.

Para o diagnóstico inicial da febre alta associada à artralgia, foi solicitada a sorologia para dengue (negativa) e chicungunha (positiva). Para o edema de membros inferiores, foi solicitado um eco-Doppler colorido, que evidenciou trombo de aspecto subagudo em veia poplítea direita.

Com relação ao tratamento da trombose de veia poplítea, optou-se por iniciar um anticoagulante (AC) oral direto que não necessitasse de AC parenteral associado, visto que a enoxaparina, único AC parenteral disponível na instituição, está relacionado à trombocitopenia induzida por heparina. Portanto, foi iniciada apixabana na dose de 10 mg de 12-12h por sete dias e, posteriormente, 5 mg de 12-12h.

O paciente melhorou progressivamente dos sintomas e recebeu alta com analgésico oral (paracetamol) e apixabana, a princípio por três meses.

## DISCUSSÃO

As infecções virais podem estar associadas a um processo inflamatório sistêmico agudo e crônico, que acometem especialmente o sistema osteoarticular, levando, muitas vezes, a um quadro de artrite inflamatória que, eventualmente, pode se cronificar^3^. Na FC, aproximadamente 48% dos pacientes mantêm um quadro de artrite por pelo menos seis meses^2^^-^ 4. Esse fato é, provavelmente, corroborado pela elevação persistente de marcadores inflamatórios, como a interleucina-6^2^.

A associação entre infecções por vírus da imunodeficiência humana (HIV), hepatite C e influenza, e o tromboembolismo venoso (TEV) já é bem estabelecida^5^^-^ 7. Diversos mecanismos podem explicar isso, como o aumento sérico dos fatores pró-coagulantes, por lesão e ativação endotelial, que provoca um aumento do fator tissular na membrana celular e ativação da via extrínseca da coagulação; ativação de micropartículas que atuam como catalizadores da coagulação; diminuição sérica dos anticoagulantes naturais (antitrombina e proteínas C e S da coagulação); aumento dos níveis séricos de anticorpos antifosfolipídios e do fator de von Willebrand; e aumento da adesão plaquetária, entre outros mecanismos^5^.

Embora não haja referência na literatura sobre a associação entre o CHIKV e a trombose venosa profunda (TVP) e/ou a embolia pulmonar (EP), devemos ficar atentos à possibilidade do TEV se manifestar nos casos mais severos da infecção pelo CHICV, pois o processo inflamatório agudo e crônico, que pode levar à lesão endotelial, associado à imobilização dos pacientes pela poliartralgia intensa e pela astenia, e à desidratação secundária à febre alta, poderiam servir de fatores desencadeantes para o desenvolvimento de TVP e/ou EP em paciente portador, ou não, de trombofilia. No caso relatado acima, o paciente havia sido investigado ambulatorialmente, sem sucesso, para trombofilia devido ao histórico de TVP em seu filho durante a adolescência, alguns anos antes, em que se confirmou a presença de fator V de Leiden, em sua forma heterozigótica, mas este fora herdado de sua mãe. Betancur et al. relatam um caso de paciente portadora de lúpus eritematoso sistêmico e anticorpos antifosfolipídios (anticoagulante lúpico e anticardiolipina) que foi a óbito após infecção por CHIKV, que deflagrou a forma catastrófica da síndrome do anticorpo antifosfolipídio^8^.

Outro fato ao qual devemos estar atentos é que, após a introdução do CHIKV no continente americano em 2013, houve um aumento progressivo do diagnóstico de FC, com 59.932 casos confirmados nos 44 países e territórios das Américas^2^. Esse fato pode explicitar de forma mais clara se existe relação entre CHIKV e TVP e/ou EP.
