# Supplementary material for: Trombose venosa profunda e vírus chicungunha
Source: J Vasc Bras. 2017 Jan-Mar;16(1):60–2. [Article in Portuguese] doi: 10.1590/1677-5449.009616 (PMC5829695; doi:10.1590/1677-5449.009616)
Supplement: Translation

# Translation: Deep venous thrombosis and chikungunya virus

**Authors:** Marcos Arêas Marques, Fernanda Penza Adami de Sá, Otília Lupi, Patricia Brasil, Arno von Ristow

**Affiliations:** 1 Universidade do Estado do Rio de Janeiro – UERJ, Departamento de Angiologia, Rio de Janeiro, RJ, Brazil.; 2 Hospital Universitário Pedro Ernesto, Departamento de Angiologia, Rio de Janeiro, RJ, Brazil.; 3 Fundação Oswaldo Cruz – FIOCRUZ, Instituto Nacional de Infectologia Evandro Chagas, Rio de Janeiro, RJ, Brazil.; 4 Pontifícia Universidade Católica do Rio de Janeiro – PUC-Rio, Departamento de Cirurgia Vascular, Rio de Janeiro, RJ, Brazil.

**Keywords:** chikungunya virus, chikungunya fever, deep venous thrombosis

## Abstract

Some systemic viral infections can be linked to development of deep venous thrombosis and/or pulmonary embolism. This association has already been well described in patients infected by human immunodeficiency virus (HIV), hepatitis C, and influenza. The chikungunya virus is the etiologic agent of chikungunya fever and it has recently been introduced to the American continent. As yet, there is no firm foundation for a relationship between chikungunya and thromboembolism, but the progressive increase in its incidence, the fact that this infection very often causes severe locomotion restrictions due to polyathralgia, and the possibility of direct endothelial injury suggest that cases of venous thromboembolism may begin to be described. In this case report, we describe a patient who developed thrombosis of the right popliteal vein after being admitted for treatment of severe polyathralgia and fever caused by chikungunya virus infection.

## INTRODUCTION

Chikungunya fever (CF) is caused by the chikungunya virus (CHIKV), an *alphavirus* transmitted to humans via bites of mosquitoes of the *Aedes* genus that was first described in the 1950s in central Africa and later in other countries in Africa and Asia.^1^ In 2007, CHIKV arrived in Europe, causing a CF outbreak in Italy,^1^ and this probably provoked the movement of CHIKV in the direction of new territories, such as Australia and the Western Hemisphere.^1^ In December of 2013, the Pan American Health Organization published an alert on autochthonous transmission of CHIKV in the Americas for the first time.^2^

Clinically, CF is characterized by sudden onset of high fever (> 38.9 °C), shivering, and photophobia that generally persist for seven days, associated, in the majority of cases, with severe polyathralgia, usually symmetrical, involving hips, elbows, fingers, knees, and ankles, and limiting patients’ locomotion for months.^1^^,^ 2

Occasionally, patients may exhibit maculopapular rash on the trunk, face, and extremities, itching, headaches, tiredness, nausea, vomiting, conjunctivitis, cervical lymphadenopathy, and myalgia.^1^^,^ 2 Laboratory findings of CF include acute leukopenia, thrombocytopenia, hypokalemia, and mildly elevated hepatic transaminases.^1^ The most common acute complications are secondary to central nervous system involvement, such as Guillain-Barré syndrome, or ocular effects, such as uveitis, and retinitis.^1^ Chronically, development of joint syndromes is common, with persistent polyarthritis in 30-40% of cases.^2^ Vascular involvement in CF has been described little and is generally restricted to persistent Raynaud phenomenon after the acute phase.^1^ Here, we describe the case of a patient who developed thrombosis of the right popliteal vein while in hospital after admission for treatment of CF and severe polyathralgia.

## CASE DESCRIPTION

A 55-year-old, Caucasian, male, ex-smoker presented at a walk-in center with fever (> 39 °C), asthenia, headaches, and arthralgia in right knees and shoulder, with onset 3 days previously. He was discharged from hospital with instructions to maintain hydration and a prescription for paracetamol. Three days later he returned to the walk-in center complaining that the high fever, asthenia, and headaches had not abated and the arthralgia had increased in intensity and spread to involve the ankles, and now also had pain in the left calf and bilateral edema of the ankles, more obvious on the left. Emergency laboratory tests revealed moderate thrombocytopenia (108,000 platelets), leukopenia (3,800 leukocytes), and normal hematocrit (41%). In view of the intensity of the fever and polyathralgia and the asymmetrical edema in lower limbs, the patient was admitted for control of symptoms and diagnostic investigations.

Serology tests for dengue (negative) and chikungunya (positive) were requested in response to the initial presentation of high fever combined with arthralgia. Color Doppler ultrasonography examination was conducted to investigate the lower limb edema and showed a thrombus with subacute appearance in the right popliteal vein.

It was decided to treat the popliteal vein thrombosis with a direct oral anticoagulant (AC) that did not require a parenteral AC in combination, since enoxaparin, which was the only parenteral AC available at the institution, is linked to heparin-induced thrombocytopenia. Apixaban was therefore started at a dosage of 10 mg every 12h for seven days and then reduced to 5 mg every 12h.

The patient progressively improved from his symptoms and was discharged on oral analgesic (paracetamol) and apixaban, initially for 3 months.

## DISCUSSION

Viral infections can be accompanied by acute and chronic systemic inflammatory processes, which involve the osteoarticular system in particular, and which very often leads to inflammatory arthritis that can sometimes become chronic.^3^ In approximately 48% of CF patients, arthritis continues for at least 6 months.^2^^-^ 4 This state is likely to be confirmed by persistently elevated inflammatory markers, such as interleukin-6.^2^

The association between human immunodeficiency virus (HIV), hepatitis C, and influenza infections and venous thromboembolism (VTE) has already been well-established.^5^^-^ 7 There are several mechanisms that could explain this, such as increased of serum levels of procoagulatory factors, endothelial injury and activation, which provokes increased expression of tissue factor in cell membranes and activation of the extrinsic coagulation pathway; activation of microparticles that act as coagulation catalysts; reduction of serum levels of natural anticoagulants (antithrombin and C and S coagulation proteins); increased serum levels of antiphospholipid antibodies and von Willebrand factor; and increased platelet adhesion, among other mechanisms.^5^

Although there are no references in the literature describing associations between CHIKV and deep venous thrombosis (DVT) and/or pulmonary embolism (PE), we should be aware of the possibility that VTE could manifest in more severe cases of infection by CHICV, since the acute and chronic inflammatory processes, which can cause endothelial injury, combined with immobilization of patients by intense polyathralgia and asthenia and dehydration secondary to high fever, could prove to be trigger factors for development of DVT and/or PE in patients who have (or do not have) thrombophilia. In the case described above, the patient had been investigated clinically, unsuccessfully, for thrombophilia because his son had had DVT in adolescence some years before, and in whom factor V Leiden had been detected, in its heterozygous form, inherited from his mother. Betancur et al. describe the case of a patient with systemic lupus erythematosus and antiphospholipid antibodies (lupus anticoagulant and anticardiolipin) who died after a CHIKV infection that triggered antiphospholipid antibody syndrome with catastrophic results.^8^

Another fact that should be considered is that there has been a progressive increase in diagnoses of CF since CHIKV was introduced to the Americas in 2013, with 59,932 confirmed cases in the 44 countries and territories of the Americas.^2^ This fact may help to elucidate whether there is indeed a relationship between CHIKV and DVT and/or PE.
